# Assessment of lipid response to acute olanzapine administration in healthy adults

**DOI:** 10.1002/edm2.119

**Published:** 2020-02-28

**Authors:** Avital Nahmias, Priska Stahel, Satya Dash

**Affiliations:** ^1^ Department of Medicine Banting & Best Diabetes Center University of Toronto Toronto ON Canada; ^2^ University Health Network Toronto ON Canada

**Keywords:** atypical antipsychotics, dopamine, lipid metabolism, olanzapine

## Abstract

**Background:**

Atypical antipsychotics (AAP) can induce hypertriglyceridaemia and type 2 diabetes. Weight gain contributes to these effects, but there is evidence that AAP can have acute metabolic effects on glycaemia independent of weight change.

**Aims:**

We undertook a single‐blind crossover study in eight healthy volunteers to assess whether the AAP olanzapine acutely increases triglyceride and free fatty acid in response to a high‐fat oral load (50 g fat with no carbohydrate) and whether these effects are attenuated by the dopamine D2 receptor agonist bromocriptine.

**Methods:**

Participants underwent three treatments in random order: Olanzapine 10 mg plus placebo (OL + PL), Olanzapine 10 mg plus bromocriptine 5 mg (OL + BR) and placebo plus placebo (PL + PL).

**Results:**

Olanzapine increased plasma prolactin, an effect that was reversed by co‐administration of the D2 receptor agonist bromocriptine (*P* = .0002). There were no significant differences in postprandial triglyceride (*P* = .8), free fatty acid (*P* = .4) or glucose (*P* = .8).

**Conclusion:**

These results suggest that AAPs likely do not directly increase postprandial lipids but may do so indirectly via changes in body weight and/or glycaemia.

## Graphical abstract

Atypical antipsychotics are causally linked to gysglycaemia and dyslipidaemia. These effects are in part mediated by weight gain, but prior evidence suggests that these drugs may have direct effects on glucose acutely, in the absence of weight gain. Here, we assessed the acute effects of olanzapine on postprandial lipids, in the absence of dysglycaemia and weight gain. We found no acute effects of this atypical antipsychotic. This suggests that the dyslipidaemic effects are likely mediated by weight gain and/or dysglycaemia.

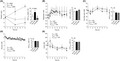

## 1 INTRODUCTION

Insulin resistance and type 2 diabetes are associated with increased triglyceride (TG)‐rich lipoproteins in the fasted and fed state. There is considerable evidence that postprandial hypertriglyceridaemia is a major cardiovascular risk factor.(1, 2) Atypical antipsychotics (AAPs) such as olanzapine are effective therapies for schizophrenia and psychosis but increase the risk for insulin resistance, type 2 diabetes and hypertriglyceridaemia.(3) Weight gain, a recognized adverse effect of AAPs, is likely a major contributor to these adverse metabolic effects. However, there is evidence that AAPs can have acute metabolic effects independent of weight gain. A single dose of olanzapine prior to a frequently sampled intravenous glucose tolerance test in healthy volunteers decreased glucose effectiveness, raised fasting glucose and decreased fasting free fatty acids.(4) The mechanisms underlying these effects are not fully elucidated but D2 receptor antagonism may contribute. Notably, bromocriptine, a D2 receptor agonist, is an approved treatment for type 2 diabetes and reduces plasma TG.(5, 6)

The acute effects of AAPs, independent of weight gain, on postprandial lipids have not been established. Further, whether these effects are dependent on acute changes in glycaemia and insulin sensitivity has not been determined. In addition, the role of D2 receptor antagonism in these potential metabolic effects is unknown. In this study, we assessed the acute effects of olanzapine on postprandial TG and free fatty acid in healthy, lean volunteers. As carbohydrate ingestion and changes in postprandial insulin can affect circulating lipids,(7, 8, 9) we assessed the acute effect of a 50‐g fat load with negligible carbohydrate and protein. This study design enabled us to evaluate the acute effect of olanzapine independent of weight change and acute changes in glycaemia. We also investigated whether co‐administration of bromocriptine, a dopamine (D2) receptor agonist, attenuated/reversed any acute effects of olanzapine.

## 2 STUDY DESIGN AND METHODS

The study was approved by the Institutional Review Board of University Health Network in accordance with the Declaration of Helsinki. All participants gave written, informed consent.

Healthy adults with no co‐morbidities or prescription medications were recruited (Table 1) in this single‐blind crossover study with 3 visits in random order separated by 2‐4 weeks: Olanzapine 10 mg plus placebo (OLA + PL), Olanzapine 10 mg plus bromocriptine 5 mg (OLA + BR) and placebo plus placebo (PL + PL). Following an overnight fast, subjects ingested a high‐fat drink providing 50 g of fat with no carbohydrate and protein (Calogen, Nutricia). Concomitantly, subjects were given oral olanzapine/bromocriptine/placebo treatments. Following treatment administration, hourly BP and pulse rate measurements were taken. Blood samples were collected at regular intervals for 8 hours after treatment. Samples were centrifuged and plasma extracted and stored at −20°C until further analysis. Plasma was analysed for glucose, TGs and free fatty acids by enzymatic assay. Plasma insulin and serum prolactin were analysed by radioimmunoassay and a chemiluminescent detection (Abbott Architect), respectively.

**Table 1:** Demographic and physiological characteristics of participants at screening

Age (years)	50.1 ± 4.0
BMI (kg/m^2^)	24.4 ± 0.7
Ethnicity	7 Caucasian/1 African‐American
Sex (Male/Female)	7/1
Fasting glucose (mmol/L)	5.1 ± 0.2
Creatinine (umol/L)	80.1 ± 4.3
eGFR (ml/min/1.73 m^2^)	95.6 ± 4.9
Cholesterol (mmol/L)	5 ± 0.3
Fasting TG (mmol/L)	1 ± 0.12
ALT (U/L)	17.4 ± 3.0
AST (U/L)	21.8 ± 1.9
ALP (U/L)	67.1 ± 8
TSH (mIU/L)	1.7 ± 0.2

Abbreviations: ALP, alkaline phosphatase; ALT, alanine aminotransferase; AST, aspartate transaminase; BMI, body mass index; eGFR, estimated glomerular filtration rate; TG, triglycerides; TSH, thyroid stimulating hormone.

We recruited thirteen participants, of whom eight subjects completed all visits with generation of data. Of the remaining five participants, 1 did not fit study inclusion criteria and was withdrawn, 1 withdrew consent and 2 were lost to follow‐up after their screening visit. A 5th participant completed 1 study visit only with administration of placebo/placebo. Here, we have presented data on the eight participants who completed all three study visits.

Incremental area under the curve was calculated by the trapezoid method for data normalized to baseline and analysed by PROC GLM with Tukey's adjustment (SAS version 9.4, SAS Institute Inc). Time courses were analysed by PROC MIXED of SAS with time as a repeated measure, where time, treatment and treatment × time interaction were considered fixed effects and participants were considered random effects. First‐order autoregressive covariance structure was assumed. All data are expressed as mean ± SEM.

## 3 RESULTS

**Figure 1:**
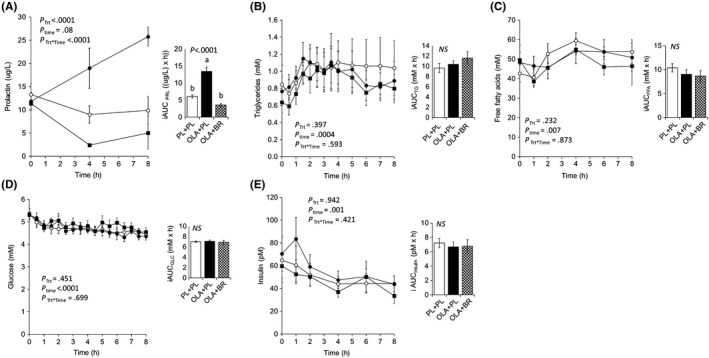
Plasma hormones and metabolites following a high‐fat drink with concomitant administration of placebo (PL + PL), olanzapine 10 mg (OLA + PL) or olanzapine 10 mg and bromocriptine 5 mg (OLA + BR). (A) prolactin; (B) triglycerides; (C) free fatty acids; (D) glucose; and (E) insulin

Baseline plasma prolactin (PL: 13.3 ± 2.4 ug/L; OLA: 11.8 ± 2 ug/L; OLA + BR: 11.4 ± 1.1 ug/L), TG (PL: 0.9 ± 0.2 mmol/L; OLA: 0.8 ± 0.2 mmol/L; OLA + BR: 0.6 ± 0.1 mmol/L), FFA (PL: 42.7 ± 4.5 mmol/L; OLA: 48.3 ± 5.2 mmol/L; OLA + BR: 49.5 ± 7.7 mmol/L), glucose (PL: 5.4 ± 0.2 mmol/L; OLA: 5.3 ± 0.2 mmol/L; OLA + BR: 5.3 ± 0.3 mmol/L) and insulin (PL: 65 ± 20 pmol/L; OLA: 71 ± 16 pmol/L; OLA + BR: 60 ± 11 pmol/L) did not differ between treatments. Olanzapine increased plasma prolactin, an effect that was reversed by co‐administration of the D2 receptor agonist bromocriptine (*P* = .0002), suggestive of D2 receptor–mediated activity—prolactin iAUC (incremental area under the curve) µg/L × hour: PL + PL 6.1 ± 0.5 vs OL + PL 13.4 ± 1.22 vs OL + BR 3.5 ± 0.4, *P* < .0001) (Figure 1A). There were no significant differences between treatments in postprandial triglyceride (iAUC mmol/L × hour, PL + PL: 9.7 ± 0.9, OL + PL: 10.4 ± 0.7, OL + BR: 11.6 ± 1.25) (Figure 1B), free fatty acid (iAUC mmol/L × hour PL + PL: 10.4 ± 0.9, OL + PL 9.03 ± 0.9, OL + BR: 8.7 ± 1.1) (Figure 1C), glucose (iAUC mmol/L × hour PL + PL: 7 ± 0.2, OL + PL: 7.1 ± 0.2, OL + BR: 6.9 ± 0.4) (Figure 1D) or insulin (iAUC pmol/L × hour PL + PL: 7.2 ± 0.7, OL + PL: 6.7 ± 0.7, OL + BR: 6.8 ± 0.9) (Figure 1E). When sequence of treatment was added to the PROC GLM model for AUCs, the *P*‐values for sequence were .436, .456 and .657 for triglycerides, glucose and free fatty acids, respectively.

Adverse effects: Three participants experienced transient symptomatic hypotension with olanzapine administration.

## 4 DISCUSSION

Chronic olanzapine use has been implicated in weight gain, dyslipidaemia, insulin resistance and type 2 diabetes.(10) However, it is not clear to what extent the lipid and other metabolic abnormalities are due to weight gain versus direct acute effects of olanzapine. Data from animal studies suggest that direct metabolic effects of olanzapine, independent of weight gain, contribute.(11) A single dose of olanzapine rapidly and robustly decreased insulin sensitivity in rats, as indicated by decreased glucose infusion rate and peripheral glucose utilization as well as increased hepatic glucose production in the setting of a hyperglycaemic‐euglycemic clamp. In the hyperglycaemic clamp setting, olanzapine impaired beta‐cell insulin secretion.(11) Two‐week subchronic olanzapine treatment induced dyslipidaemia in rats despite no excess weight gain. This was associated with increased expression of lipogenic genes in liver and adipose tissue.(12)

Here, we evaluated the effect of a single dose of olanzapine compared to placebo and olanzapine and bromocriptine on plasma lipids after ingestion of a high‐fat liquid. We hypothesized that acute olanzapine treatment would increase postprandial TG with attenuation by co‐administration with bromocriptine treatment suggesting a D2 receptor–mediated effect. Olanzapine increased prolactin, an effect reversed with bromocriptine, strongly indicating that the doses of olanzapine and bromocriptine used in this study had D2 receptor–mediated biological effects. However, we did not observe any change in TG or free fatty acid level. Notably, a 9th participant completed only 1 study visit. We also analysed the data with inclusion of data from this visit and found no significant difference in plasma TG and FFA. The data suggest that at this dose, acute olanzapine administration does not directly affect lipid metabolism and that acute D2 receptor modulation likely has no acute effects on postprandial lipids in humans.

The dose of olanzapine used in this study is used therapeutically and is equivalent to the daily dose used in other studies. It is possible that more prolonged administration and/or higher olanzapine dosing are needed to generate a measurable effect on lipid metabolism. Eight days of olanzapine (10 mg) treatment in the absence of weight gain worsened insulin‐mediated glucose disposal and blunted the insulin‐induced decline of plasma free fatty acids and TG concentrations, despite a reduction in FFA.(13) Trials involving healthy volunteers with 10 mg/d olanzapine administration, ranging between 8 and 21 days of medication exposure, have yielded inconsistent results with some suggesting changes in insulin sensitivity,(13, 14, 15, 16) whereas others have failed to find an association(17, 18) and/or reported early increases in weight thought to account for the changes in glucose metabolism.(17) Furthermore, these studies have not always accounted for changes in fat distribution, which can occur without significant weight changes.

This study was designed to assess the acute effect of olanzapine independent of changes in glycaemia, and therefore, we used a high‐fat liquid with negligible carbohydrate and protein. As expected, no changes were seen in plasma glucose across treatments. Further studies are needed to assess the acute response to a mixed macro‐nutrient meal following administration of olanzapine.

The inherent limitations of small number of subjects and a single dose design preclude definitive conclusions; however, our study did not show a direct effect of olanzapine on lipid metabolism, and thus favours prolonged weight gain and/or dysglycaemia as a major mechanism for dyslipidaemia observed in patients receiving atypical antipsychotic drugs.

## CONFLICT OF INTEREST

SD has received consulting fees and speaker fees from Eli Lilly and Novo Nordisk.

## AUTHOR CONTRIBUTIONS

SD designed the experiments. P.S and AN performed data analyses and prepared the manuscript. SD edited the manuscript.

## ETHICS STATEMENT

This study was approved by the Research Ethics Board, University Health Network and Health Canada.

## Data Availability

Data sharing is not applicable to this article as no additional data were created or analysed in this study.
